# Remdesivir in treating hospitalized patients with COVID-19: A renewed review of clinical trials

**DOI:** 10.3389/fphar.2022.971890

**Published:** 2022-09-08

**Authors:** Zhenchao Wu, Zhifei Han, Beibei Liu, Ning Shen

**Affiliations:** ^1^ Department of Respiratory and Critical Care Medicine, Peking University Third Hospital, Beijing, China; ^2^ Center for Infectious Diseases, Peking University Third Hospital, Beijing, China; ^3^ School of Basic and Clinical Medicine, Shandong First Medical University, Jinan, China

**Keywords:** remdesivir, COVID-19, SARS-CoV-2, clinical trials, efficacy, safety

## Abstract

Since December 2019, COVID-19 has spread across the world almost through 2.5 years. As of 16 June 2022, the cumulative number of confirmed cases of COVID-19 worldwide has reached 542.62 million, and the death toll has risen to 6.33 million. With the increasing number of deaths, it is urgent to find effective treatment drugs. Remdesivir, an investigational broad-spectrum antiviral drug produced by Gilead has been shown to inhibit SARS-CoV-2, *in vitro* and *in vivo*. This review is aimed to analyze the feasibility of remdesivir in COVID-19 and put forward the shortcomings of present clinical studies. We systematically searched PubMed and Web of Science up until 24 May 2022, using several specific terms such as “remdesivir” or “GS-5734” and “COVID-19” or “SARS-CoV-2” and retrieved basic researches and clinical studies of remdesivir in COVID-19. In this review, we summarized and reviewed the mechanism of remdesivir in SARS-COV-2, clinical trials of using remdesivir in COVID-19, analyzed the efficacy and safety of remdesivir, and judged whether the drug was effective for the treatment of COVID-19. In different clinical trials, remdesivir showed a mixed result in the treatment of COVID-19. It seemed that remdesivir shortened the time to recovery and had an acceptable safety profile. However, more clinical trials are needed to test the efficacy and safety of remdesivir.

## Introduction

COVID-19 was first identified as an emerging infectious disease in December 2019, and then raging around the world. As of 16 June 2022, there are 542.62 million infections and over 6.33 million deaths reported by Worldometer (2022), which are continuing to rise daily. To date, many countries around the world are struggling to prevent, control and treat COVID-19. SARS-COV-2, a β-coronavirus causing COVID-19, has four key proteins: spike protein (S), envelope protein (E), membrane protein (M), and nucleocapsid protein (N). Among them, the S protein could bind to the ACE2 receptor of human beings to infect host cells (Hillen et al., 2020; Jackson et al., 2022). Scientists reported that SARS-CoV-2 is a positive-stranded single-stranded RNA coronavirus and its genome is approximately 30 kb (Zhu et al., 2020). Recently, several major studies showed that high risk of infection, virulence of virus variants, clinical manifestations of early stage, severity of illness condition, inflammatory cytokine storm, short and long-term prognosis are all related to viral load, which reveals that virus replication is the key point to infection and pathogenesis of novel coronavirus (Chen et al., 2021; Drayman et al., 2021). Based on available evidence, it is self-evident that RNA virus RNA-dependent RNA polymerase (RdRp) is the key factor to novel coronavirus replication (Wang et al., 2020a; Gao et al., 2020; Hillen et al., 2020; Yin et al., 2020). Hillen et al. (2020) elaborated the cryo-electron microscope structure of RdRp which includes viral proteins nonstructural protein 12 (nsp12), nsp8, and nsp7, and positive and negative strands of viral double-stranded RNA molecules. The active-site cleft of nsp12 binds to the first turn of RNA and mediates the activity of RdRp with conserved residues. Two copies of nsp8 bind to the opposite side of the cleft and locate the second round of RNA. The long spiral extension in nsp8 protrudes along with the existing RNA, forming positively charged “sliding poles.” This provides a basis for exploring RdRp as a binding target for COVID-19 therapeutics (Hillen et al., 2020).

**FIGURE 1:**
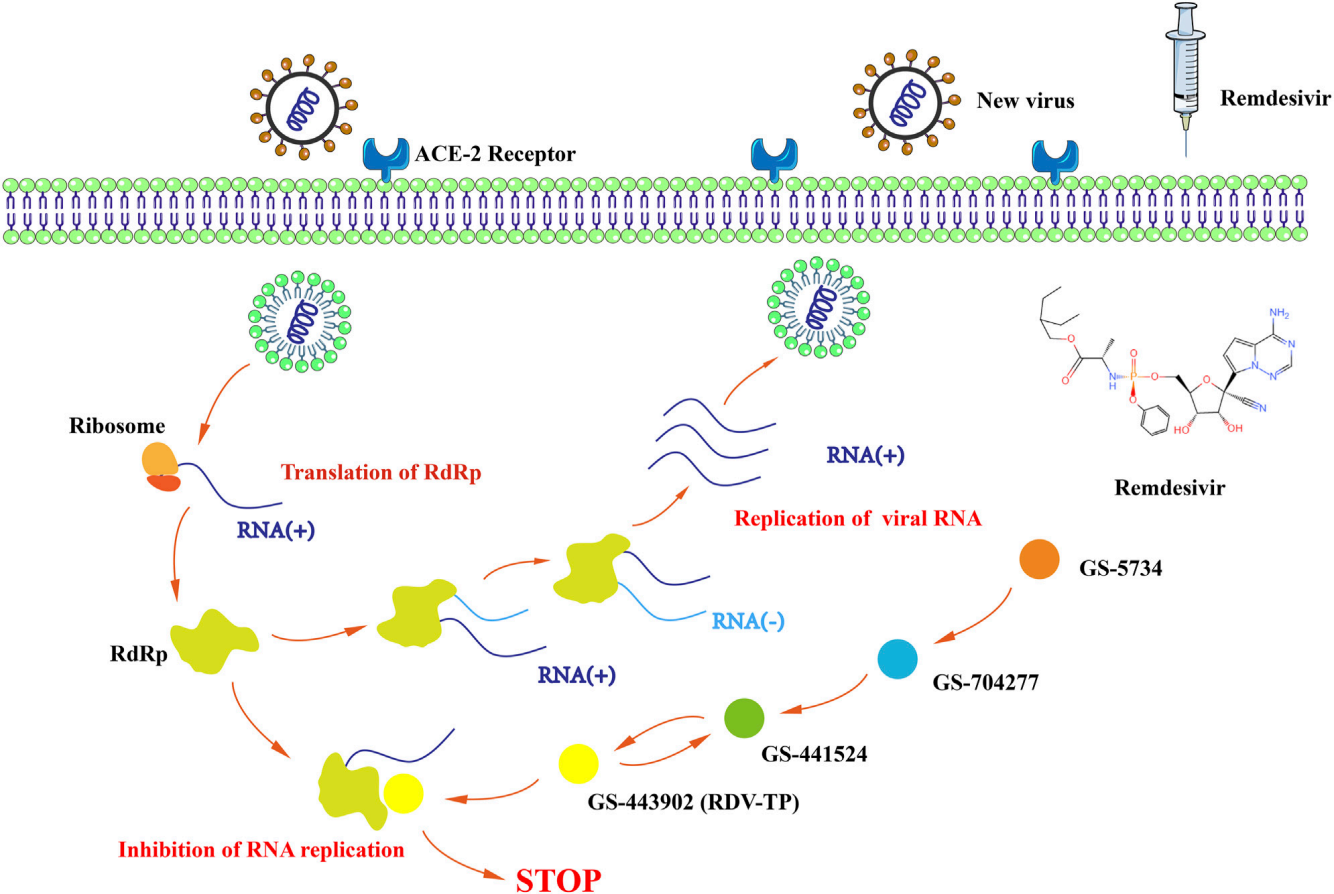
The mechanism by which remdesivir inhibits the replication of SARS-COV-2.

Remdesivir (RDV, formerly GS-5734) is a nucleoside analog with broad-spectrum anti-RNA virus activity developed by Gilead, which was previously developed as a drug to fight the Ebola virus (Mulangu et al., 2019). Remdesivir is a phosphoramidite prodrug of 1′-cyano adenosine analogs. The metabolic rate of remdesivir *in vivo* is faster than most antivirus drugs (plasma half-life, T½ 1 h), and the intermediate metabolites of remdesivir, GS-441524 and so on, have a long actuation duration of the drug (plasma half-time, T½ 24.5 h), which make the plasma concentration of remdesivir reach and remain at an efficiency level immediately. Remdesivir has low renal excretion (<10%) while radiolabeled GS-441524 has a 49% dose in urine (Humeniuk et al., 2020; Jorgensen et al., 2020). Remdesivir could generate a variety of metabolites to inhibit the replication of SARS-COV-2 *in vivo* and *in vitro*. Inside cells, GS-5734 rapidly converted into GS-704277, followed that GS-704277 converted into GS-441524, and then into GS-443902. Dephosphorylation of nucleoside monophosphate analogs produces GS-441524, an intermediate metabolite of RDV, gets activated through various kinases responsible for initial phosphorylation including the adenosine kinase (ADK) that is moderately expressed in all tissues. Some studies suggest GS-441524 might be a better candidate to treat COVID-19 *in vivo* and *in vitro* (Tempestilli et al., 2020; Yan and Muller, 2020; Brunotte et al., 2021; Cox et al., 2021; Wei et al., 2021; Yan et al., 2021; Li et al., 2022; Sukeishi et al., 2022). For example, the combination of GS-441524 and fluoxetine had well tolerance and displayed antiviral effects against SARS-CoV-2 variants *in vitro* (Brunotte et al., 2021; Schloer et al., 2021). It is metabolized in the cell to produce an active triphosphate form -NTP, which could inhibit RNA-dependent RNA polymerases (RdRps) of SARS-COV-2 (Elfiky, 2020; Gordon et al., 2020; Shannon et al., 2020; Yan and Muller, 2020). Enzyme kinetics indicated that this RdRp efficiently incorporates the active triphosphate form of RDV (RDV-TP, GS-443902) into RNA (Humeniuk et al., 2020; Jorgensen et al., 2020). Incorporation of RDV-TP at position i caused the termination of RNA synthesis at position i+3, which could inhibit the replication of RNA of SARS-COV-2 (Gordon et al., 2020) (Figure 1). Unlike the Ebola virus, for treatment of which remdesivir was initially designed, SARS-COV-2 has a mismatch reparation enzyme, ExoN, which eventually excises the incorporated remdesivir nucleoside from the viral RNA, thus rescuing the replicating complex. However, with the presence of pibrentasvir, RNA terminated with the intermediate metabolites of remdesivir was largely protected from excision by the ExoN (Shannon et al., 2020; Wang et al., 2022). Recent studies found that GS-621763 (the prodrug of GS-441524) administration could reduce viral load and improve pulmonary function in a COVID-19 mice and ferrets model (Cox et al., 2021; Schafer et al., 2021; Schafer et al., 2022). These results may suggest that remdesivir might have a robust effect on COVID-19 treatment.

Since the first successful use of remdesivir to treat COVID-19 in the United States on 31 January 2020 (Holshue et al., 2020), clinical scientific research teams around the world have launched different trials to verify the possibility of remdesivir in COVID-19 from the perspectives of efficacy and safety. These trials have sparked contentious debates. Recently, due to the effect of drug combination with remdesivir and the recommendation by authorities (FDA, NIH, WHO) anew, remdesivir (ren min de xi wang, people’s hope, a transliteration of chinese) was placed in high hope once more. Therefore, we review the most important clinical trials of COVID-19 in the world since its outbreak, try to interpret them with various viewpoints and leave more inspiration.

We used several specific search terms such as “remdesivir” or “GS-5734” and “COVID-19” or “SARS-CoV-2” and retrieved clinical trials including case reports and RCTs on the treatment of COVID-19 by remdesivir on PubMed, Web of Science. We reviewed several large-scale RCTs with important clinical significance to analyze the safety and efficacy of remdesivir in COVID-19. Remdesivir has shown different effects in different clinical trials with COVID-19 patients. The following will be combined with these studies to analyze the current performance of remdesivir in clinical applications from two aspects: the efficacy and safety of remdesivir.

## The efficacy of remdesivir in COVID-19

### Efficacy in case reports of COVID-19

On 31 January 2020, the Washington State 2019-nCoV Case Investigation Team reported the diagnosis and treatment process of the first case treated with remdesivir in the United States. The 35-year-old male patient was diagnosed with COVID-19 by positive SARS-COV-2 nasopharyngeal and oropharyngeal swab testing with real-time reverse transcriptase-polymerase chain reaction (rRT-PCR) on 20 January 2020, and then was sent to the isolation ward for treatment. In addition to persistent fever and dry cough, the patient suffered from obvious digestive symptoms, including nausea and vomiting diarrhea and abdominal discomfort, at the early stage of hospitalization, without any shortness of breath or chest pain or abnormal vital signs. Subsequently, feces were used to detect the presence of SARS-COV-2. On the fifth day, the patient developed an acute pulmonary infection. After the administration of the antibiotics -vancomycin and cefepime, doctors considered remdesivir to inhibit the key enzyme RdRp of SARS-COV-2 replication and decided to draw support from remdesivir for experimental treatment. In the next 2 days, the patient’s condition improved significantly. As of 30 January 2020, the patient was afebrile and only had a slight cough (Holshue et al., 2020). Two months later, Angela Haczku et al. from the University of California gave remdesivir within 36 h to a 40-year-old female patient diagnosed with critical COVID-19. After 10 days of therapy, her condition had significantly improvement such as ventilator support requirements reducing, blood oxygen levels rising, alleviation of pulmonary inflammation, and no obvious drug adverse event. COVID-19 PCR was negative on the 12th day. After 2 weeks, the mechanical ventilation could be evacuated. Then the patient was discharged without supplemental O_2_ needed (Sanville et al., 2020). The efficacy and safety of remdesivir in the two COVID-19 patients suggested that further large-scale clinical trials could be carried out.

### Efficacy in randomized controlled trials

Remdesivir’s efficacy in COVID-19 appeared to have had mixed results in nine randomized controlled trials (RCTs) conducted around the world over the past 3 years (Table 1).

**TABLE 1:** RCTs of remdesivir in COVID-19 (RCTs online published as of 10 May 2022).

Author, Online time	Trial name, number	Study design	Country	Patients	Amount, randomization	Intervention	Control	Primary endpoint	Conclusion
Cao et al. 29 April 2020 [Wang et al., (2020b)]	NCT04257656	Randomized, double-blind, placebo- controlled, multicentre trial	China	Adults with laboratory-diagnosed SARS-CoV-2 infection, with an interval from symptom onset to enrolment ≤12 days, SaO_2_ ≤ 94% or PaO_2_/FiO_2_ ≤ 300 mm Hg, and radiology-confirmed pneumonia.	237, 2:1 (158 to remdesivir and 79 to placebo)	Remdesivir (200 mg on day 1 followed by 100 mg on days 2–10 in single daily infusions)	The same volume of placebo infusions for 10 days	Time to clinical improvement up to day 28 (6-point ordinal scale)	Remdesivir was not associated with a difference in time to clinical improvement (HR 1.23 [95% CI 0.87–1.75]).
22 May 2020. [Beigel et al., (2020)]	ACTT-1, NCT04280705	Double-blind, randomized, placebo- controlled trial	United States	Hospitalized adults with lower respiratory tract infection and COVID-19	1,062, 1:1 (541 assigned to remdesivir and 521 to placebo)	Remdesivir (200 mg loading dose on day 1, followed by 100 mg daily for up to 9 additional days)	Placebo for up to 10 days	The time to recovery (discharge from the hospital or hospitalization for infection control purposes) with an eight-category ordinal scale	Remdesivir was not associated with a difference in time to clinical improvement (HR 1.23 [95% CI 0.87–1.75]).
27 May 2020. [Goldman et al., (2020)]	The first SIMPLE trial, NCT04292899	Randomized, open-label, phase 3 trial	Multi-countries	Hospitalized patients with diagnosed COVID-19, SaO_2_ ≤ 94%.	397, 1:1 (200 patients for 5 days and 197 for 10 days)	Remdesivir (200 mg loading dose on day 1, followed by 100 mg daily for 4 additional days)	200-mg loading dose on day 1, followed by 100 mg daily for 9 additional days	Clinical status on day 14 (a 7-point ordinal scale)	There was no significant difference between a 5-day course and a 10-day course of remdesivir in severe COVID-19 patients not requiring mechanical ventilation.
21 August 2020. [Spinner et al., (2020)]	The second SIMPLE trial, NCT04292730	Three-arm randomized, open-label trial	Multi-countries	Hospitalized patients with moderate COVID-19 confirmed by PCR within 4 days of randomization.	596, 1:1:1 (197 for 10 days, 199 for 5 days, 200 for standard care)	Remdesivir (200 mg on day 1 followed by 100 mg/d)	Standard care	Clinical status on day 11 (a 7-point ordinal scale)	There was no statistically significant difference in clinical status compared with standard care
15 October 2020. [Consortium et al., (2021)]	Solidarity trial, ISRCTN83971151, NCT04315948	Large, simple, multi-country, open-label randomized trial	Multi-countries	Hospitalized patients with a diagnosis of COVID-19 and without contra-indication to any study drug.	11266, not available	Remdesivir (200 mg on day 0 followed by 100 mg/d on day 1–9)	Other study drugs	In-hospital mortality in the 4 comparisons of each study drug vs. its controls	Remdesivir had little or no effect on hospitalized COVID-19 including overall mortality, initiation of ventilation and duration of hospital stay.
11 December 2020. [Kalil et al., (2021a)]	ACTT-2, NCT04401579	Double-blind, randomized, placebo-controlled trial	United States	Hospitalized adults with COVID-19.	1,033, 1:1 (515 to combination treatment, 518 to control)	Remdesivir (200 mg loading dose on day 1, followed by a 100 mg dose on day 2–10) plus Baricitinib (4 mg daily dose for 14 days)	Remdesivir plus placebo (the same doses and days as the combination group)	The time to recovery	Baricitinib combined with remdesivir was better than remdesivir alone in reducing recovery time and accelerating improvement in clinical status among COVID-19 patients.
14 September 2021. [Ader et al., (2022)]	EudraCT2020-000936-23, NCT04315948	Phase 3, open-label, adaptive, multicentre, randomized, controlled trial	Europe	Hospitalized patients with laboratory-diagnosed SARS-CoV-2 infection.	857, 1:1 (429 to remdesivir plus standard of care and 428 to standard of care only)	Remdesivir (200 mg intravenous infusion on day 1, followed by once daily, 1-h infusions of 100 mg up to 9 days)	Standard care	The clinical status at day 15 (seven-point ordinal scale of the WHO Master Protocol)	There was no clinical benefit that the use of remdesivir in COVID-19 patients with more than 7 days symptoms, and required oxygen support.
22 December 2021. [Gottlieb et al., (2022)]	NCT04501952	Randomized, double-blind, placebo-controlled trial	United States	Patients with COVID-19 within the previous 7 days and at least one risk factor for disease progression.	562, 1:1 (279 patients in the remdesivir group and 283 in the placebo group)	Remdesivir (200 mg on day 1 followed by 100 mg on days 2 and 3)	Placebo (200 mg on day 1, 100 mg on days 2 and 3)	A composite of COVID-19 related hospitalization or death from any cause by day 28 and any adverse event.	A 3-day remdesivir course with acceptable safety and led to an 87% lower risk of hospitalization or death than placebo
22 February 2022. [Ali et al., (2022)]	CATCO (Canadian Treatments for COVID-19), NCT04330690	Pragmatic, multicentre randomized controlled trial	Canada	Hospitalized patients with COVID-19	1,282, 1:1 (634 to remdesivir plus standard care, 648 to standard care alone)	Remdesivir (200 mg intravenously on day 0, followed by 100 mg daily) plus standard care	Standard care	In-hospital mortality	There was no difference in mortality between two groups.

On 29 April 2020, Bin Cao and his colleagues selected 237 eligible patients with severe COVID-19 at ten hospitals in Hubei, China for the first randomized, double-blind, placebo-controlled multicenter trial (NCT04257656). Eligible patients were randomly assigned to the remdesivir group (158 people) and the placebo group (79 people) at a ratio of 2:1. Patients were given an intravenous injection of remdesivir (200 mg on day 1, 100 mg on day 2–10, once a day infusion) or an equivalent placebo infusion for 10 consecutive days, allowing patients to use lopinavir-ritonavir, interferon and corticosteroids at the same time. The primary endpoint was clinical improvement time from randomization to day 28, regardless of the point of a decline of two levels on a six-point ordinal scale of clinical status (from 1 = discharged to 6 = death) or discharge came first. Then a preliminary intention-to-treat (ITT) population analysis and drug safety analysis were performed. The final research results showed that the use of remdesivir had nothing to do with the time difference in clinical improvement (HR 1.23, 95% CI 0.87–1.75). Although not statistically significant, with symptoms lasting 10 days or less, patients who received remdesivir improved clinically faster than patients who received placebo [RR 1.52 (0.95–2.43)] (Wang et al., 2020c).

On 22 May 2020, Beigel et al. (2020) led an international, randomized, placebo-controlled phase 3 clinical trial (ACTT-1, NCT04280705), which evaluated remdesivir plus standard treatment for 10 days used in adult hospitalized patients with mild/moderate to severe COVID-19. A total of 1,063 patients participated in the randomization of a 1:1 ratio (541 patients in the remdesivir group and 522 patients in the placebo group). The medication regimen was an intravenous infusion of remdesivir or placebo 200 mg loading dose on day 1, followed by a daily intravenous infusion of remdesivir or placebo 100 mg maintenance dose from day 2 to day 10 or until to patients were discharged/died. The primary outcome was the time to recovery assessed on the eight-category ordinal scale (from 1 = not hospitalized and no limitations of activities to 8 = death). The preliminary results of the trial reported in May showed that the recovery time of patients in the remdesivir group was shorter than that of the placebo group (median of 11 days in the remdesivir group and 15 days in the placebo group) and the speed of recovery was faster. The conclusion report in October showed the treatment efficacy of remdesivir on COVID-19, consistent with the preliminary trial results in May. It was confirmed that compared with placebo plus standard treatment, remdesivir plus standard treatment significantly shortened the patient’s recovery time within a 29-day cycle. The results demonstrated the median recovery time in the remdesivir group was 10 days and that the median recovery time was 15 days in the placebo group. At the same time, the recovery rate of the remdesivir group was increased by 29% compared with that of the placebo group (RR 1.29; 95% CI 1.12–1.49; *p* < 0.001) (Beigel et al., 2020). Recently, ACTT-2 reported a clinical trial by using a combination treatment of remdesivir (≤10 days) and baricitinib (≤14 days) in COVID-19 patients on 11 December 2020. The primary outcome was the time to recovery. The results showed that Baricitinib plus remdesivir was superior to remdesivir alone in reducing recovery time (the median recovery time: the combination treatment group 7 days vs. the control group 8 days, *p* = 0.03) (Kalil et al., 2021b).

On 27 May 2020, Goldman et al. (2020) from Gilead reported the first SIMPLE study (GS-US-540- 5773) (a randomized, open-label, phase 3 trial). A total of 397 patients from eight regions were randomized in a 1:1 ratio to receive a 5-day course of remdesivir (*n* = 200) or a 10-day course of remdesivir (*n* = 197). All patients received 200 mg remdesivir on day 1 followed by 100 mg/d. The primary endpoint was the clinical status on day 14, based on a 7-point ordinal scale (from 1 = death to 7 = not hospitalized). The results showed that there was no significant difference between a 5-day course and a 10-day course of remdesivir in patients with severe COVID-19 not requiring mechanical ventilation (Goldman et al., 2020). Subsequently, Spinner et al. from Gilead reported the second SIMPLE study (a three-arm randomized, open-label phase 3 trial) on 15 Sep 2020. A total of 596 patients were randomized in a 1:1:1 ratio to receive a 10-day course of remdesivir (*n* = 197), a 5-day course of remdesivir (*n* = 199), or standard care (*n* = 200). Remdesivir was dosed intravenously at 200 mg on day 1 followed by 100 mg/d. The primary endpoint was the clinical status on day 11 on a 7-point ordinal scale (from 1 = death to 7 = not hospitalized). On day 11, patients in the 5-day remdesivir group had a much higher odds of a better clinical status distribution than those who received standard care (odds ratio, 1.65; 95% CI 1.09–2.48; *p* = 0.02). However, there was no significant difference in the distribution of clinical status on day 11 between the 10-day remdesivir group and the standard care groups (*p* = 0.18) (Spinner et al., 2020). Meanwhile, Olender et al. (2021) reported a secondary analysis of GS-US-540–5773 study data (remdesivir treatment group) and compared it to the GS-US-540–5807 study data (nonremdesivir-treatment group). The study has analyzed the result of 14-day recovery rate, 14-day mortality, and 28-day mortality and found that remdesivir might improve the recovery rate and decrease death odds in COVID-19 patients (Olender et al., 2021).

On 15 October 2020, WHO reported the mortality in COVID-19 patients who were respectively treated with four antiviral drugs (remdesivir, hydroxychloroquine, lopinavir-ritonavir, and interferon), which was called the Solidarity trial. A total of 11,266 patients from 405 hospitals in 36 countries/regions were randomized to receive remdesivir (2750), hydroxychloroquine (954), lopinavir-ritonavir (1,411), interferon (2063) and nonstudy drugs (4088). The study divided the corresponding controls according to each therapeutic strategy. A total of 1,253 deaths were disclosed in the report. The death rate ratios were as follows: the remdesivir group 12.5% vs. the control group 12.7%, the hydroxychloroquine group 10.2% vs. the control group 8.9%, the lopinavir group 9.7% vs. the control group 10.3%, the interferon group 12.9% vs. the control group 11.0%. The data showed that the four study drugs did not have any definite effects on mortality (*p* > 0.05), either in the overall group or in the subgroups (Consortium et al., 2021). On 22 February 2022, Ali et al. (2022) and his team led a pragmatic, multicenter randomized controlled trial (Canadian Treatments for COVID-19, CATCO), which was a substudy of the Solidarity trial. A total of 1,282 hospitalized patients with COVID-19 were randomized into two groups at a 1:1 ratio (634 to remdesivir plus standard care, 648 to standard care alone). The dose of remdesivir was 200 mg intravenously on day 0, followed by 100 mg daily. The endpoint was in-hospital mortality. The result did not show a difference in mortality between the two groups while remdesivir had a modest but significant effect on the need for mechanical ventilation (Ali et al., 2022). As a latest renew review, we always pay attention to the curative effect of remdesivir in the treatment of COVID-19. Some reports were released by related organizations in recent time. A solidarity RCT conducted by WHO reported its final results about Remdesivir and three other drugs for hospitalized patients with COVID-19, which showed remdesivir has no significant effect on patients with COVID-19 under mechanical ventilation. Among other hospitalized patients, it has a small effect on death or progression to ventilation (WHO Solidarity Trial Consortium, 2022).

On 14 September 2021, Ader et al. (2022) reported a phase 3, open-label, adaptive, multicenter, randomized, controlled trial (NCT04315948). A total of 857 adult patients from Europe were randomized in a 1:1 ratio to receive remdesivir (200 mg intravenous infusion on day 1, followed by once daily, 1-h infusions of 100 mg up to 9 days) plus standard of care and standard of care only. The primary endpoint was the clinical status on day 15. The outcome showed that there was no clinical benefit being observed from the use of remdesivir in COVID-19 patients who were symptomatic for more than 7 days, and required oxygen support (Ader et al., 2022).

On 22 December 2021, Gottlieb et al. (2022) reported a randomized, double-blind, placebo-controlled trial (NCT04501952) to research early remdesivir in preventing progression to severe COVID-19 outpatients. A total of 562 non-hospitalized patients were randomized in a 1:1 ratio to receive remdesivir (200 mg on day 1 followed by 100 mg on days 2 and 3) and placebo. The efficacy primary endpoint was a composite of COVID-19-related hospitalization or death from any cause by day 28 and the safety endpoint was any adverse event. The outcome of a 3-day course of remdesivir had an acceptable safety profile and led to an 87% lower risk of hospitalization or death than placebo (Gottlieb et al., 2022).

In terms of combination treatment of remdesivir, baricitinib plus remdesivir was better than remdesivir alone in reducing recovery time and serious adverse events (Kalil et al., 2021b). Compared with placebo plus remdesivir, Tocilizumab plus remdesivir could not shorten discharge time in severe COVID-19 patients (Rosas et al., 2021). The monoclonal antibody LY-CoV555 plus remdesivir showed no efficacy in hospitalized COVID-19 patients without end-organ failure when compared to standard treatment (including remdesivir) (Group et al., 2021). Interferon β-1a (IFN β-1a) plus remdesivir did not show better therapeutic effect than remdesivir alone in hospitalized COVID-19 patients (Kalil et al., 2021a). The combination treatment of tocilizumab plus remdesivir showed similar efficacy in severe COVID-19 patients who were treated by tocilizumab plus hydroxychloroquine (Sarhan et al., 2022). Treatment with adalimumab plus remdesivir and dexamethasone was not better than remdesivir plus dexamethasone in severe COVID-19 patients (Fakharian et al., 2021).

### The safety and side-effects of remdesivir in COVID-19

The safety of remdesivir is divided into seven parts (five systems and two special populations) to describe respectively. (See the Supplementary Appendix for more information about the adverse events).

### Systemic adverse events

The first RCT program in China (NCT04257656) conducted by Prof. Cao illustrated that the most common systemic adverse events in the remdesivir group and placebo group were hypoalbuminemia (13% vs. 15%), hypokalemia (12% vs. 14%), anemia (12% vs. 15%) and thrombocytopenia (10% vs. 6%) (Wang et al., 2020c). In ACTT-1, the most common systemic adverse events occurring in at least 5% of all patients included decreased hemoglobin levels, decreased lymphocyte counts, anemia, pyrexia, hyperglycemia, and increased blood glucose levels. The incidence of these adverse events was generally similar in the remdesivir and placebo groups (Beigel et al., 2020). In ACTT-2, the most common grade 3 or 4 adverse events occurring in at least 5% of all patients were hyperglycemia, anemia, and decreased lymphocyte count. The incidence of these adverse events was similar in the two treatment groups (Kalil et al., 2021b). In the second SIMPLE study, nausea was more common in the remdesivir group than in the standard care group (Spinner et al., 2020). In Gottlieb’s trial, the incidence of all systemic adverse events in the remdesivir group was lower than that in the placebo group, especially pyrexia (0.4% vs. 3.9%) and dizziness (1.8% vs. 3.5%) (Gottlieb et al., 2022).

### Safety in the cardiovascular system

In Cao’s RCT, cardiovascular adverse events were less frequent, and the incidence of these adverse events was similar in the two groups (Wang et al., 2020c). In ACTT-1, the incidence of atrial fibrillation and deep vein thrombosis was highest in the remdesivir group and placebo group, which were 0.9% vs. 1.9% and 1.5% vs. 2.7%, respectively. The incidence of other cardiovascular AEs was extremely similar in the two groups (Beigel et al., 2020). In ACTT-2, the incidence of atrial fibrillation in the treated group was relatively higher than that in the control group, which meant that the combination of baricitinib and remdesivir was likely to reduce the risk of atrial fibrillation (Kalil et al., 2021b). In other RCTs, there was no record of cardiovascular AEs, which was incomplete to some degree (Goldman et al., 2020; Spinner et al., 2020; Consortium et al., 2021; Olender et al., 2021; Ader et al., 2022; Ali et al., 2022; Gottlieb et al., 2022).

### Safety in the respiratory system

In Cao’s RCT, there were more patients suffering from respiratory failure or acute respiratory distress syndrome in the remdesivir group than in the placebo group (10% vs. 8%). One patient was diagnosed with recurrence of COVID-19 in the remdesivir group (Wang et al., 2020c). In ACTT-1, 47 (8.8%) serious respiratory failure adverse events were observed in the remdesivir group, including acute respiratory failure and the need for endotracheal intubation, compared with 80 (15.5%) in the placebo group. Two patients (0.4%) were diagnosed with a recurrence of COVID-19, compared with five patients in the placebo group (Beigel et al., 2020). In ACTT-2, the incidence of serious respiratory failure including acute respiratory failure and respiratory failure was similar (8.7% vs. 9.9%) (Kalil et al., 2021b). In the first SIMPLE study, the most common respiratory adverse event was acute respiratory failure (6% in the 5-day group vs. 11% in the 10-day group) (Goldman et al., 2020). In Florence’s RCT, the incidence of serious respiratory adverse events including acute respiratory failure, respiratory failure or acute respiratory distress syndrome, and pulmonary embolism (7% vs. 9%, 9% vs. 9%, 2% vs. 3% respectively) was relatively high (Ader et al., 2022). In a recent RCT of Gottlieb, there were only four mild adverse events including pneumonia (0.7% vs. 2.8%), dyspnea (2.5% vs. 5.3%), cough (3.6% vs. 6.4%) and anosmia (3.2% vs. 2.1%), which was likely due to decreased virulence of mutated SARS-COV-2 (Gottlieb et al., 2022).

### Safety in the urinary system

In the early days when remdesivir was used to treat Ebola, toxicological studies on rhesus monkeys showed that with 5, 10, and 20 mg doses (significantly higher than the EUA (Emergency Use Authorization) dose) for 7 days (Administration, 2020), the kidneys were damaged. However, in a clinical trial at that time, it did not show obvious damage to the human kidney. It can be seen that the dose has a great impact on the trial (Mulangu et al., 2019). In a prospective clinical study, remdesivir was safe for use in patients with end-stage renal disease (ESRD), and there were no serious adverse reactions. For patients with new coronary pneumonia with ESRD, remdesivir could be considered as a treatment option to reduce the patient’s recovery and discharge time (Aiswarya et al., 2021). Cao’s clinical trial showed that remdesivir did not increase the risk of renal damage in patients with new coronary pneumonia (Wang et al., 2020c). In ACTT-1, the most common urinary adverse event is an increased blood creatinine level, which was generally similar in the remdesivir and placebo groups (Beigel et al., 2020). In ACTT-2, baricitinib plus remdesivir was more likely to reduce the incidence of acute kidney injury than remdesivir alone (Kalil et al., 2021b). In clinical practice for the treatment of 103 patients with new coronary pneumonia, remdesivir treatment in the first 15 days did not show nephrotoxicity, and only two patients had grade 3 hepatotoxicity. Although remdesivir metabolites are mainly passed out of the body through the kidneys, they show the same clearance rate as normal patients in patients with impaired renal function. Therefore, it is not believed that poor renal function will cause clinical symptoms within 5 days after treatment. The related blood drug concentration increases, which leads to increased toxicity or efficacy (Grein et al., 2020). In Florence’s RCT, there was a similar incidence of acute kidney injury and kidney failure between the remdesivir group and the control group (3% vs. 4%, 1% vs. 1%) (Ader et al., 2022).

### Safety in the digestive system

In Cao’s RCT, the most common digestive events were constipation (14% vs. 15%) and increased aspartate aminotransferase (5% vs. 12%), which meant that remdesivir was likely to reduce the incidence of constipation (Wang et al., 2020c). In ACTT-1 and ACTT-2, the incidence of all digestive adverse events in the remdesivir group was higher than that in the placebo group which was likely to indicate the safety of remdesivir in the digestive system (Beigel et al., 2020; Kalil et al., 2021b). In the first SIMPLE study, the most common digestive adverse events were nausea (10% vs. 9%), increased ALT (6% vs. 8%), and constipation (6% vs. 7%) (Goldman et al., 2020). In Spinner’s clinical trial, nausea was more common in the remdesivir groups than in the standard care group (Spinner et al., 2020). In the Pamoja Tulinde Maisha (PALM) study, among 175 Ebola patients who received remdesivir, liver toxicity was not recorded as a serious adverse event. These are the data on early remdesivir treatment of Ebola (Mulangu et al., 2019). In ACTT-1, it was mentioned in the safety of the kidneys that 2 of 103 COVID-19 patients have developed grade 3 hepatotoxicities (Beigel et al., 2020). In another case study, none of the five patients treated with remdesivir had chronic liver disease. After treatment, there was no adverse reaction that could result in the development of liver damage, nor did they develop severe liver damage. It also did not cause liver failure. Although SARS-CoV-2 infection itself could cause an increase in aminotransferase, 4 of these 5 patients had normal or slightly elevated AST/ALT levels at the beginning of remdesivir treatment, indicating that remdesivir had a direct role in hepatotoxicity (Zampino et al., 2020). It is also worth noting that in a case study, a male patient with new coronary disease developed acute hepatotoxicity after receiving remdesivir and p-glycoprotein (P-gp) inhibitor treatment, which may result from the two drugs interacting with each other, and it was recommended to use P-gp with caution in the clinical treatment of remdesivir (Leegwater et al., 2021).

### Safety in children

At present, there was little data on the treatment of children with COVID-19 with remdesivir. On 25 June 2020, a nationwide observational study was conducted on children under 16 years of age with COVID-19 by the sympathetic treatment of remdesivir in Spain. During all patients receiving medication, liver enzymes were monitored every 2 or 3 days. None of the patients had elevated liver enzymes. One of the patients developed multifactorial renal damage due to multiple organ failures and nephrotoxic drugs. No clinical or other laboratory toxicity was observed (Mendez-Echevarria et al., 2021). On 5 May 2021, Goldman et al. (2021) reported compassionate use of remdesivir in children with severe COVID-19. Seventy-seven hospitalized patients (<18 years old) received remdesivir intravenously through a compassionate-use program between March 21 and 22 April 2020. The results showed that the safety of remdesivir was well-tolerated, with a low risk of serious adverse events (16%). Most adverse events were related to COVID-19 or comorbid conditions. Laboratory abnormalities, including elevations in transaminase levels, were common and mild (Goldman et al., 2021).

### Safety in pregnant women

Because remdesivir may adversely affect the fetus, pregnant patients were unlikely to be included in clinical trials. There were also fewer clinical cases of remdesivir used in pregnant women infected with COVID-19. In an early study of 5 pregnant women with severe COVID-19 using remdesivir, all five patients developed aminotransferase abnormalities (McCoy et al., 2020). In a sympathetic treatment of three pregnant patients with COVID-19 using remdesivir, the potential adverse effects of hepatitis using remdesivir were emphasized. This adverse effect is 6%–8% in the nonpregnant population, which may be related to the overlap of the causes of pregnancy-related transaminase elevation. Although only 3 cases had a limited ability to draw broad conclusions, remdesivir was well-tolerated in pregnant women and may be effective. Except for abdominal infections, no adverse reactions to remdesivir were found (Igbinosa et al., 2020). In addition, Burwick et al. (2021) and his team reported a compassionate use plan for remdesivir, which included 86 pregnant women with severe COVID-19. In the entire cohort, 29% of women had adverse events and 16% of women had serious adverse events. Among pregnant women, adverse events that occurred in at least 3% of women included anemia, constipation, deep vein thrombosis, dysphagia, unexplained hypertension, hypoxia, nausea, and pleural effusion, which reflected the signs and symptoms of pregnancy and COVID-19. Seven pregnant women discontinued the study drug due to adverse events, including five due to elevated liver enzyme levels, one due to nausea, and the other due to hemoptysis. No postpartum women had any adverse events leading to the discontinuation of remdesivir. A 30-year-old woman who received remdesivir after childbirth died postpartum due to severe acute respiratory distress syndrome and related cytokine storms; this death was attributed by the treating clinician to potential COVID-19, rather than remdesivir (Burwick et al., 2021). Recently, there was a case report about remdesivir and human milk. A 28-year-old primipara after delivery 2 days who was diagnosed with COVID-19, and then she was given remdesivir. The remdesivir concentration in maternal serum and breast milk was measured, and the ratio of drug concentration in milk to serum was low (0.089), as was the relative infant dose (0.0070%), which suggested that breastfeeding was safe during treatment with remdesivir and can be expected to protect the infant from infection (Wada et al., 2022).

Summarily, considering that pregnant women and the general population have a similar risk of contracting the new coronavirus, the higher risk of serious infection, including death, and a potential risk of vertical transmission to the fetus, there is an urgent need to evaluate the treatment of COVID-19 in such patients (Chen et al., 2020; Sentilhes et al., 2020; Norman et al., 2021). However, current clinical studies have shown that remdesivir treatment does not result in more serious adverse reactions in pregnant women with COVID-19 than in nonpregnant patients.

## Discussion

Remdesivir, an antiviral drug, although originally designed and developed to fight the Ebola epidemic, was once considered the hope of treating patients suffering from COVID-19. RDV arrests the progression of the RdRp/RNA replicative complex of SARS-COV-2. The first RCT organized by Cao’s team showed that there was no difference in clinical symptoms between remdesivir and placebo treatment after 28 days, but the symptoms tended to improve within the 10 days. Then a phase III clinical solidarity trial led by the WHO indicated that 2,750 people in the remdesivir group received the drug for 10 days. It did not reduce the mortality rate of COVID-19 patients, and it was not found that the drug shortened the hospitalization time of patients. However, a week later, remdesivir was the first drug approved by the U.S. Food and Drug Administration (FDA, 2022) for the treatment of hospitalized patients with COVID-19 over 12 years old, which was likely due to the efficacy of treating adults with COVID-19 in ACTT-1 (Rubin et al., 2020). On 21 April 2022, WHO updated the ninth COVID-19 treatment guidelines in the British Medical Journal. They suggested treatment with remdesivir, for patients with nonsevere COVID-19 at the highest risk of hospital admission because recent RCTs, especially Gottlieb’s, have shown that remdesivir was a benefit of reducing the hospitalization rates, and as the risk of hospitalization increased, the drug effect was better (WHO, 2022). To date, nine RCTs involving 17, 292 people with remdesivir in the treatment of COVID-19 have shown that remdesivir may reduce the recovery time of adult patients with nonsevere COVID-19, which suggested that remdesivir should be used in the early stage of infection. In terms of safety, there was no significant difference in the probability of adverse events between the remdesivir group and the control group in clinical trials (Beigel et al., 2020; Wang et al., 2020b; Goldman et al., 2020; Spinner et al., 2020; Consortium et al., 2021; Ader et al., 2022; Ali et al., 2022; Gottlieb et al., 2022). Remdesivir does not increase the risk of kidney injury in patients, but it could cause an abnormal increase in transaminase. The remdesivir metabolite GS-441524 has been reported to have a better bioactivation and pharmacodynamics and kinetics than the parenteral remdesivir. Considering the combination treatment, remdesivir- itraconazole and remdesivir- fluoxetine showed a potent efficacy for anti-SARS-COV-2 in COVID-19 animals (Brunotte et al., 2021; Schloer et al., 2021). While the combination therapy of remdesivir and baricitinib was superior to remdesivir alone in terms of shortening the patient’s recovery time and accelerating the improvement of the clinical status. Baricitinib plus remdesivir had a lower incidence of serious adverse events than remdesivir alone (Kalil et al., 2021b). Although the combination of the two drugs could be considered in clinical treatment, actually, the data of combination with remdesivir were not unsatisfied. There was no predictably significant difference in the efficacy and safety of other drugs combined with remdesivir, compared with the control group (Kalil et al., 2021a; Fakharian et al., 2021; Group et al., 2021; Rosas et al., 2021; Sarhan et al., 2022). Furthermore, combined medications carry the risk of drug-drug interactions that may lead to a reduced therapeutic benefit or even severe adverse effects. The combination of remdesivir and p-glycoprotein (P-gp) inhibitor has the risk of drug-drug interactions that lead to a reduced therapeutic benefit or even severe adverse effects (Leegwater et al., 2021).

For children, remdesivir did not cause any severe adverse events in most cases (Goldman et al., 2021; Mendez-Echevarria et al., 2021). Recently, FDA updated the guideline for the treatment of COVID-19. As the first approved drug to remediate the COVID-19 outbreak in children. This drug usage covered children under the age of 12-year-old, at least 28 days or older and weighing at least 3 kg, who were with positive results of novel coronavirus. These children have mild to moderate COVID-19 with hospitalization or not, who are likely to develop into severe cases even hospitalization or death (FDA, 2022). In addition, researchers did not suggest an increased risk of severe disease among pregnant women, the risk of vertical infection is not increased or slightly increased in newborn babies (Chen et al., 2020; Sentilhes et al., 2020; Norman et al., 2021). However, at present, the evidence of remdesivir in treating pregnant women with COVID-19 is still quite limited, especially in terms of drug safety. A study reported the concentration of remdesivir metabolite GS-441524 in maternal serum was 33.1–389.9 ng/ml and in human milk was 13.50–284.9 ng/ml. The half-lives of GS-441524 in maternal serum and milk were 25.1 and 9.3 h respectively (Wada et al., 2022). Therefore, it is urgent to evaluate the efficacy of remdesivir in pregnant patients, which requires more clinical trials.

Some of the above studies suggested remdesivir improved clinical outcomes in hospitalized patients with moderate-to-severe COVID-19. Whether the use of remdesivir in outpatients with COVID-19 at high risk for disease progression prevents hospitalization is uncertain. A recent study about early remdesivir administration in outpatients with COVID-19, which showed among non-hospitalized patients who were with COVID-19 progression at high risk, a 3-day course of remdesivir had an acceptable safety profile and resulted in an 87% lower risk of hospitalization or death than placebo (Gottlieb et al., 2022). It suggested that the use of remdesivir for patients with mild-to-moderate patients with COVID-19 in early infection stage, which could shorten the time to recovery, reduce hospitalization rate, mortality and improve overall prognosis. However, this phenomenon needs more RCTs to verify.

## Conclusion

Although there are some adverse events in the treatment of COVID-19, the safety of remdesivir seems to have a reliable guarantee. At the same time we recommend real-time monitoring of the liver and kidney functions of patients to prevent adverse reactions caused by remdesivir. We guess the use of remdesivir for patients with mild and early infection, which may reduce the time to recovery and hospitalization rate. However, this guess about efficacy and safety of remdesivir needs more RCTs to verify in non-hospitalized and hospitalized patients with COVID-19. In addition, it urgently needs to analyze the efficacy and safety of remdesivir in the treatment of children and pregnant women with COVID-19. In a word, we comprehensively reviewed the previous valuable studies, which may provide some instructive suggestions for the future on the treatment and research of remdesivir to COVID-19.
